# Chronic rhinosinusitis during immune checkpoint inhibition in melanoma patients

**DOI:** 10.1111/ddg.70063

**Published:** 2026-04-06

**Authors:** Yenny Angela, Sinja Sistani, Mareike Alter, Ralf Gutzmer

**Affiliations:** ^1^ Department of Dermatology Venereology Allergology and Phlebology Johannes Wesling University Hospital Minden Ruhr University Bochum Bochum Germany

Dear Editors,

Immune checkpoint inhibitors (ICIs), including programmed cell death protein 1 (PD‐1) antibodies, are a cornerstone of modern systemic cancer therapy. Although immune‐related adverse events (irAEs) involving the skin, gastrointestinal tract, and endocrine organs are well documented, upper airway involvement – particularly chronic rhinosinusitis (CRS) – has rarely been reported and is likely underrecognized. Here, we describe three patients with melanoma who developed CRS during PD‐1 inhibitor therapy.

A 71‐year‐old man was diagnosed with stage IIB melanoma (pT3b, pN0, cM0) in August 2023. Adjuvant therapy with pembrolizumab was initiated in November 2023. Two months later, he developed immune‐mediated thyroiditis resulting in hypothyroidism, which was well controlled with levothyroxine replacement therapy. In August 2024, he presented with progressive nasal obstruction, dyspnea, and a dry cough, particularly when in the supine position. The patient was a nonsmoker, with no pulmonary or atopic comorbidities. Total IgE was within the normal range (32.0 kU/l, reference < 100 kU/l), and specific IgE against common aeroallergens (house dust mite, grasses, birch, cat dander) was negative, indicating no relevant allergic sensitization.

Baseline cranial MRI (cMRI) was unremarkable (Figure 1a, b). During follow‐up, and in correlation with the clinical symptoms, cMRI revealed mucosal swelling of the paranasal sinuses and partial opacification of the mastoid air cells (Figure 1c, d). Pulmonary function tests and chest CT were normal. A diagnosis of chronic sinusitis with postnasal drip was established. Systemic corticosteroid therapy (prednisolone 20 mg/day for 7 days, 10 mg/day for 7 days, then 5 mg/day for 14 days) in combination with intranasal mometasone led to rapid clinical and radiological improvement (Figure 1e, f). Adjuvant immunotherapy was discontinued after 8 months.

**FIGURE 1:**
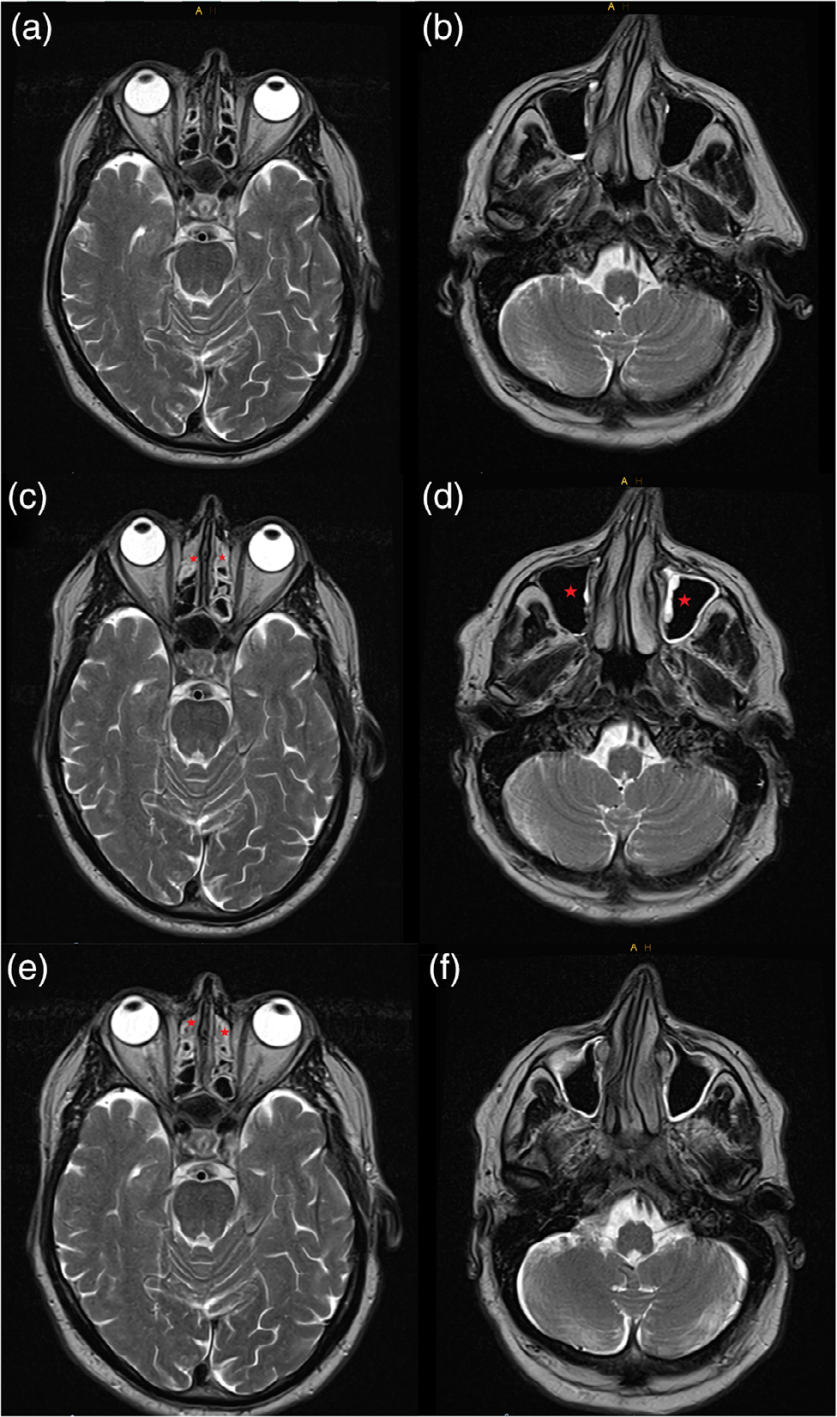
Axial T2‐weighted MRI images of Patient 1 showing (a, b) unremarkable findings prior to initiation of immune checkpoint inhibition; (c, d) symmetrical mucosal thickening of the anterior and posterior ethmoid cells under ICI therapy (red asterisks); (e, f) marked regression of inflammatory changes after systemic and topical corticosteroid therapy.

In May 2022, a 60‐year‐old male patient without comorbidities was diagnosed with melanoma of the ear with in‐transit cutaneous metastasis, stage IIIC (pT4a, pN1c, cM0). Adjuvant therapy with nivolumab was initiated in July 2022 and was initially well tolerated.

After 10 months, he developed frontal and maxillary sinus pressure, nasal discharge, and exertional dyspnea. In June 2023, cMRI demonstrated severe pansinusitis without evidence of invasive fungal infection or air‐fluid levels (Figure 2c, d), compared with only minor mucosal changes prior to immunotherapy (Figure 2a, b). Nasal endoscopy revealed hyperemic, edematous mucosa and bilateral grade 3 hypertrophy of the inferior turbinates. Laboratory findings showed total IgE of 94.9 kU/l with specific IgE to *Dermatophagoides pteronyssinus*, birch, rye, and timothy grass.

**FIGURE 2:**
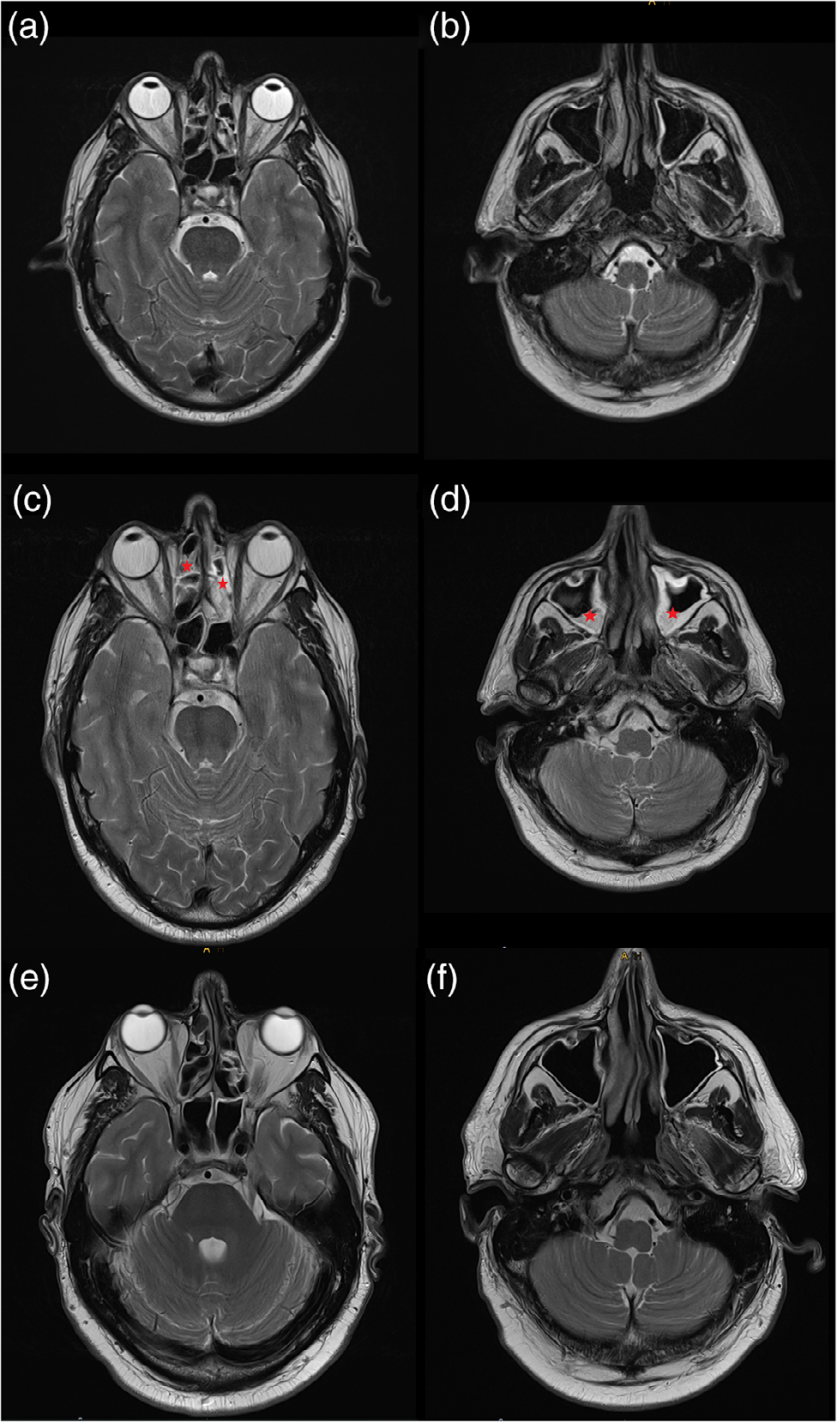
Axial T2‐weighted MRI images of Patient 2 showing (a, b) normal baseline imaging before ICI therapy; (c, d) pronounced mucosal thickening of the ethmoid sinuses with early involvement of the maxillary sinuses under ICI therapy (red asterisks); (e, f) substantial improvement following systemic corticosteroid treatment.

Oral amoxicillin/clavulanic acid had no effect. Topical antihistamine and corticosteroid sprays provided only partial improvement. Systemic prednisolone (20 mg/day with gradual tapering over a total of 3.5 months) resulted in marked clinical and radiological improvement (Figure 2e, f).

A 70‐year‐old female heavy smoker with 40 pack‐years was diagnosed with melanoma stage IIIA (pT2a, pN1a, cM0) in February 2024. She started adjuvant pembrolizumab in April 2024.

After 11 months, she developed frontal and maxillary sinus pressure, nasal obstruction, and exertional dyspnea. Endoscopy revealed inflammatory mucosal changes. cMRI demonstrated right‐sided mucosal thickening in the maxillary and ethmoid sinuses without air‐fluid levels (Figure 3c, d), compared with unremarkable baseline imaging (Figure 3a, b). Laboratory testing revealed markedly elevated total IgE levels (2,500 kU/l; reference < 100 kU/l), while specific IgE against common aeroallergens (house dust mite, grasses, birch, cat dander) was negative, indicating no evidence of type I sensitization. She was treated with intranasal mometasone spray. Pembrolizumab could be continued and completed as scheduled over 12 months. Follow‐up imaging in June 2025 showed regression of CRS‐related mucosal changes under ongoing topical corticosteroid therapy (Figure 3e, f).

**FIGURE 3:**
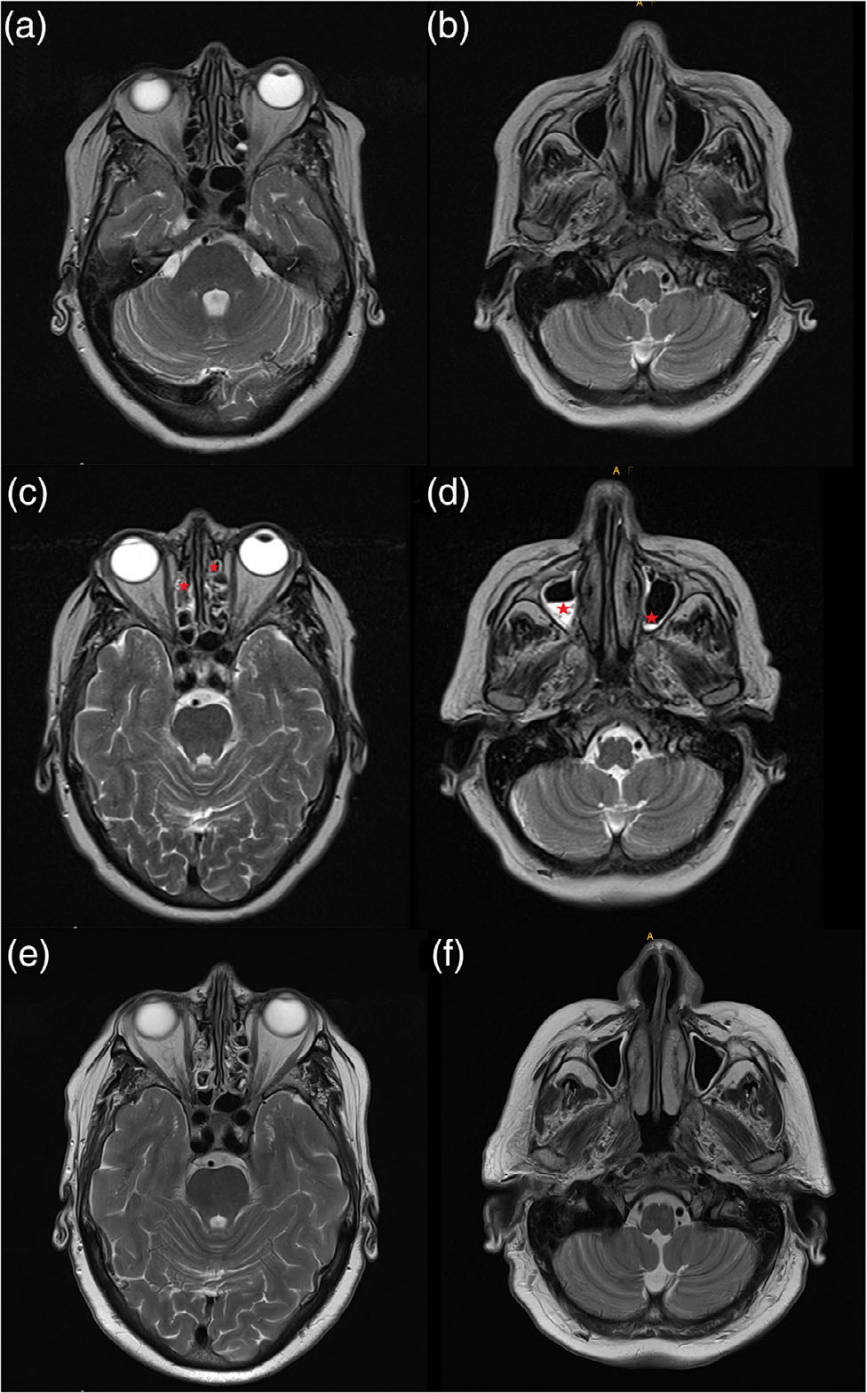
Axial T2‐weighted MRI images of Patient 3 showing (a, b) unremarkable pre‐treatment findings; (c, d) inflammatory changes in the anterior ethmoid cells during ICI therapy (red asterisks); (e, f) near‐complete resolution of mucosal thickening after topical corticosteroid therapy.

Chronic rhinosinusitis during PD‐1 inhibition represents a rare immune‐related adverse event that has so far been reported only in isolated cases or small case series. Due to its nonspecific presentation, it is likely underdiagnosed or misinterpreted. A systematic retrospective analysis detected CRS in 12 of 108 patients (11 %) treated with pembrolizumab.^1^

The latency to symptom onset in our series (9–11 months) was longer than previously reported (4–7 months).^1^, ^2^, ^3^ Clinical manifestations included sinus pressure, nasal obstruction, postnasal drip, and dyspnea. Two patients also experienced nocturnal cough related to postnasal drip. Although not life‐threatening, these symptoms can significantly impair quality of life. Differential diagnoses include infection (often with fever or purulent discharge) and immune‐mediated pneumonitis. Cranial imaging, performed routinely for oncologic surveillance, can aid in diagnosis when CRS is suspected.

Intranasal mometasone was used in all three of our patients. This was sufficient in patient 3, whereas patients 1 and 2 required additional systemic corticosteroids. The literature also describes an off‐label approach using a carboxymethylcellulose‐based foam tamponade (SINU‐FOAM^®^) soaked with triamcinolone (40 mg/ml), applied three times at 2 to 3‐week intervals, resulting in symptom improvement. This approach is anecdotal and has not been systematically evaluated; its use remains experimental.^3^

The pathophysiology of ICI‐associated CRS remains incompletely understood. One proposed mechanism is enhanced T‐cell activation following PD‐1 pathway blockade, resulting in local mucosal inflammation, as observed in other irAEs. Emerging data show increased PD‐1 and PD‐L1 expression in sinonasal polyp tissue in patients with classical CRS without prior PD‐1 inhibition.^4^ This suggests a physiological role of the PD‐1 axis in mucosal immune regulation. Wang et al. further reported enrichment of PD‐1^+^CXCR5^−^CD4^+^ T cells in polyp tissue, promoting B‐cell activation and local immunoglobulin production, particularly in eosinophilic polyps with elevated IgE levels.^5^ Reduced PD‐L1/PD‐L2 expression was also noted, indicating diminished regulatory control of these T‐cell subsets. Collectively, these findings support an immunomodulatory role of the PD‐1 pathway in sinonasal mucosa and suggest that PD‐1 inhibitor–associated CRS may result from functional shifts in mucosal T‐cell subsets with altered effector responses.^1^, ^3^

This case series highlights the need for increased clinical awareness of CRS as a rare and potentially underdiagnosed immune‐related adverse event during immune checkpoint inhibition (Table 1).

**TABLE 1:** Overview of clinical features in three cases of chronic rhinosinusitis during PD‐1‐directed immune checkpoint inhibition.

Clinical features	Patient 1	Patient 2	Patient 3
Sex	Male	Male	Female
Age	71	60	70
Stage of melanoma	IIB	IIIC	IIIA
Treatment indication	Adjuvant	Adjuvant	Adjuvant
Smoking status	Non‐smoker	Non‐smoker	Smoker
Atopic diathesis	No	Yes (house dust mite, birch, grasses)	No
ICI regimen	Pembrolizumab	Nivolumab	Pembrolizumab
Time to symptom onset	9 months	10 months	11 months
Symptoms	Nasal obstruction, dyspnea, dry cough	Sinus pressure, nasal discharge, dyspnea	Sinus pressure, nasal obstruction, dyspnea, dry cough
Treatment	Systemic corticosteroids, corticosteroid nasal spray	Systemic corticosteroids, corticosteroid nasal spray	Corticosteroid nasal spray only
CRS outcome (follow‐up)	6 months post‐ICI: stable symptom control, continued nasal spray	3 months post‐ICI: stable symptom control, continued nasal spray	3 months post‐ICI: stable symptom control, continued nasal spray

## CONFLICT OF INTEREST STATEMENT

Y.A. received honoraria for lectures/advisory boards from Roche, BMS, MSD, Novartis, Amgen, Merck Serono, Almirall Hermal, SUN, Sanofi, and Pierre Fabre and received support for congress participation from Novartis and Pierre Fabre. M.A. received honoraria for lectures and advisory board activities from Roche, BMS, MSD, Novartis, Amgen, Merck Serono, Almirall Hermal, SUN, Sanofi, Pierre Fabre, AbbVie, Janssen, Pfizer, Takeda, Incyte, and Falk and received support for congress participation from Roche, Novartis, BMS, SUN, Merck Serono, Pierre Fabre, AbbVie, and UCB. R.G. received honoraria for lectures/advisory boards from Roche, BMS, MSD, Novartis, Amgen, Merck Serono, Almirall Hermal, SUN, Sanofi, Pierre Fabre, Incyte, Immunocore, and Delcath, received research funding from Novartis, Amgen, Merck Serono, SUN Pharma, Sanofi/Regeneron, Almirall Hermal, Kyowa Kirin, and Recordati, and received support for congress participation from SUN and Pierre Fabre. S.S. declares no conflict of interest.
